# The Euler characteristic as a basis for teaching topology concepts to crystallographers

**DOI:** 10.1107/S160057672101205X

**Published:** 2022-02-01

**Authors:** Bartosz Naskręcki, Mariusz Jaskolski, Zbigniew Dauter

**Affiliations:** a Faculty of Mathematics and Computer Science, Adam Mickiewicz University, Poznan, Poland; b Department of Crystallography, Faculty of Chemistry, A. Mickiewicz University, Poznan, Poland; c Institute of Bioorganic Chemistry, Polish Academy of Sciences, Poznan, Poland; d Macromolecular Crystallography Laboratory, NCI, Argonne National Laboratory, Argonne, USA

**Keywords:** Harriot theorem, Descartes’ theorem, Euler’s polyhedral formula, modified Euler characteristic, space-filling polyhedra, asymmetric unit, Dirichlet domains

## Abstract

The simple Euler polyhedral formula, expressed as an alternating count of the bounding faces, edges and vertices of any polyhedron, *V* − *E* + *F* = 2, is a fundamental concept in several branches of mathematics. Obviously, it is important in geometry, but it is also well known in topology, where a similar telescoping sum is known as the Euler characteristic χ of any finite space. The value of χ can also be computed for the unit polyhedra (such as the unit cell, the asymmetric unit or Dirichlet domain) which build, in a symmetric fashion, the infinite crystal lattices in all space groups. In this application χ has a modified form (χ_m_) and value because the addends have to be weighted according to their symmetry. Although derived in geometry (in fact in crystallography), χ_m_ has an elegant topological interpretation through the concept of orbifolds. Alternatively, χ_m_ can be illustrated using the theorems of Harriot and Descartes, which predate the discovery made by Euler. Those historical theorems, which focus on angular defects of polyhedra, are beautifully expressed in the formula of de Gua de Malves. In a still more general interpretation, the theorem of Gauss–Bonnet links the Euler characteristic with the general curvature of any closed space. This article presents an overview of these interesting aspects of mathematics with Euler’s formula as the leitmotif. Finally, a game is designed, allowing readers to absorb the concept of the Euler characteristic in an entertaining way.

## Abstract 2

The concept of the Euler characteristic as a property of space-filling polyhedra in crystallography is discussed in a didactic way; a number of other aspects, all of fundamental importance in mathematics, are covered. A game is designed to allow the readers to absorb the concept of the Euler characteristic in an entertaining way.

## 1. Introduction

Topology, broadly defined as the study of certain properties of geometric figures (or spaces) that do not change as these figures or spaces undergo continuous deformation, is a relatively young branch of mathematics, developed as a distinct field by Henri Poincaré (see the biographical notes in Appendix *A* [App appa]) at the end of the 19th century. However, topologists usually associate the foundation of their discipline with Leonhard Euler (see Appendix *A* [App appa]), whose famous formula relating the numbers of vertices, edges and faces of any three-dimensional polyhedron, 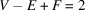 , is of fundamental importance in topology. This formula later became the basis of the concept of the Euler characteristic χ, which can be applied not only to polyhedra [more generally termed polytopes (for a definition see Appendix *B* [App appb])] but also to more unusual (to our senses) topological figures, such as spheres, toruses, strips *etc*. Roughly speaking, χ is defined as an alternating sum of the numbers of *k*-cells associated with a given figure, where 0-cells are equivalent to vertices, 1-cells to edges, and so on up to *k* = *N*, *N* being the dimension of the figure. Moreover, these concepts can be applied not only to finite objects, such as, *e.g.*, the cube, but also to objects that extend in space infinitely. An excellent example of such an infinite topological object is a crystallographic lattice: periodic and infinite in three dimensions (or in any number *N* of dimensions, 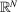 ). It was the consideration of the crystallographic unit cell (as an object sharing its bounding elements, or *k*-cells, with its neighbors) and its minimal asymmetric part, the asymmetric unit (ASU), that some time ago made us realize that the Euler’s formula for such ‘incompletely bounded’ figures will be different, yielding a sum that is smaller by 1 (Dauter & Jaskolski, 2020 ▸). This concept was extended, with proof, to the Euler characteristic, termed for such objects the modified Euler characteristic 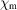 (Naskręcki *et al.*, 2021*a* ▸). A completely general treatment of 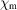 based on the topological notion of orbifolds (Appendix *B* [App appb]) (Naskręcki *et al.*, 2021*b* ▸) showed that these abstract topological ideas have very practical extension to crystallography.

In this paper we introduce in an accessible way the notions of the Euler characteristic and modified (multiplicity-weighted) Euler characteristic in relation to crystallographic polyhedra, lattices and symmetry groups. Moreover, we show how the idea of 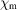 can be derived from some fundamental topological theorems, such as Harriot’s theorem or Descartes’ theorem. It is our goal to use the concept of the Euler characteristic to make topology more familiar, and useful, to crystallographers.

The article is constructed in such a way that sections that present cornerstone concepts are concluded with ‘Take-home messages’, which we hope the reader will have understood and will remember. As the finale, we have designed a game ‘Let’s compute Euler’s number’ which offers an entertaining way of absorbing the concept of the Euler characteristic.

### 1.1. A note on polyhedra and solids

We start from a Euclidean space of a given dimension *N*. In such a space we will consider sets, called *k*-cells, which are topologically equivalent to closed balls of dimension 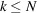 . Such cells can be joined together to form new subsets, *e.g.* forming a rectangle from four edges (1-cells) that overlap at four vertices (0-cells).

A *k*-polytope embedded in a Euclidean space of dimension *N* is a union of cells of dimensions ranging from 0 to *k*. In particular, a 2-polytope in 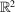 is usually called a polygon, while a 2-polytope in 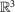 is typically an empty ‘skin’ (polyhedron), consisting of flat faces joined at straight edges and point vertices. If the points inside the boundary of this ‘skin’ are also included in the definition of the object, it becomes a solid (a 3-polytope in 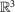 ), which formally also includes several 3-cells (interiors *I*). In this special case, the topological Euler characteristic of the solid equals one ( 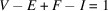 ). In particular, one might ask, what is the crystallographic unit cell? In the extreme case one might say that it is a skeleton of 12 edges of a parallelepiped and the faces do not matter. However, since the crystal unit cell is ultimately filled with concrete matter, atoms and molecules, most crystallographers would view the unit cell as a solid parallelepiped, with proper faces bounding the three-dimensional interior. In this view, the translationally repeated unit cells cover all points of the 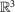 space.

Depending on the context, we will refer to a *k*-polytope (built from cells of dimensions between 0 and *k*) in 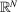 for 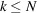 and call it *k* dimensional. The standard notation is to say that we have a polytope in 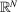 , which denotes a solid *N*-dimensional polytope. We will always make the proper distinction because, as noted above, even the adopted definition influences the result of the sum in Euler’s polyhedral formula and characteristic.

### 1.2. Plan of the paper

**Figure 1:**
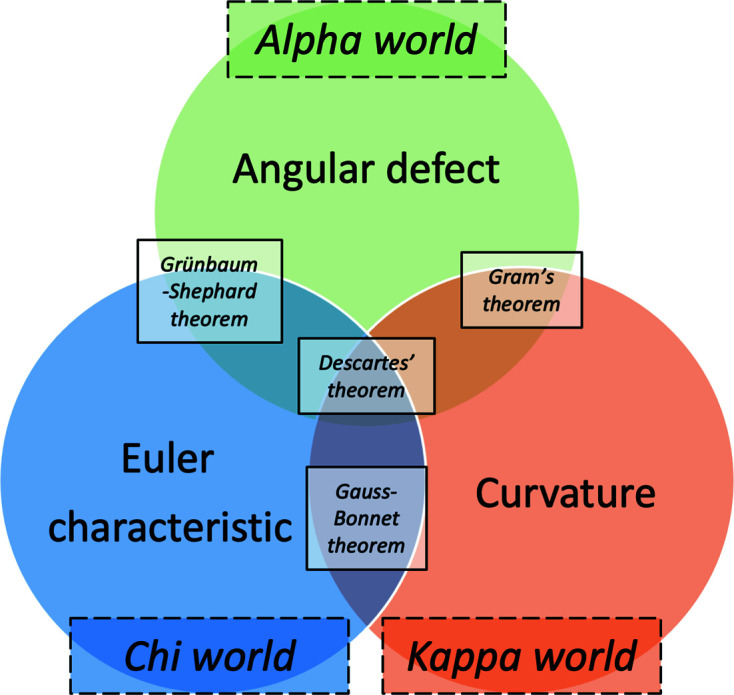
The three circles represent the three different worlds, each built around the boldface definition in that circle. Each white box represents the theorem that connects a particular set of worlds.

Our goal in this paper is to familiarize the crystallographic community in an accessible way with the broad system of concepts and theorems centered around the notion of the Euler characteristic. The concepts introduced in the following sections are connected in various ways. Fig. 1 ▸ is a concise scheme of the paper that should serve as a roadmap for readers.

We divided the concepts we introduce into three realms: the alpha world – centered on the idea of measuring angles in geometric objects; the kappa world – built around the concept of the curvature (Appendix *B* [App appb]) and global change of shape; and the chi world – concepts stemming from the notion of the Euler characteristic.

Each of these worlds has a non-trivial intersection with the other worlds. In Section 2 we discuss in detail the major concepts related to the alpha world. In Sections 3 and 4 we discuss the main properties and definitions of the Euler characteristic and its various generalizations.

In Section 5 we link the alpha and kappa worlds with the chi world via the fundamental result of Gauss (see Appendix *A* [App appa]) and Bonnet (see Appendix *A* [App appa]), which on the one hand connects the concept of curvature (kappa world) to the Euler characteristic for smooth surfaces, and on the other hand connects the total angular defect (Appendix *B* [App appb]) (alpha world) to the same Euler characteristic in a polytopal analog of the Gauss–Bonnet theorem. In Section 6 we discuss further developments and more technical points of the introduced mathematical concepts.

The paper is illustrated with several examples and exercises, including a puzzle game (in Section 3.2), which should help interested readers to gain a more thorough understanding of the concepts introduced. The concepts and theorems discussed in the following sections are summarized in Table 1 ▸. The main message of this paper is that the Euler characteristic is a simple, explicit and useful concept from topology that can be applied in crystallography to study space groups and their lattice tessellations.

## 2. Harriot theorem and the angular defect

**Figure 2:**
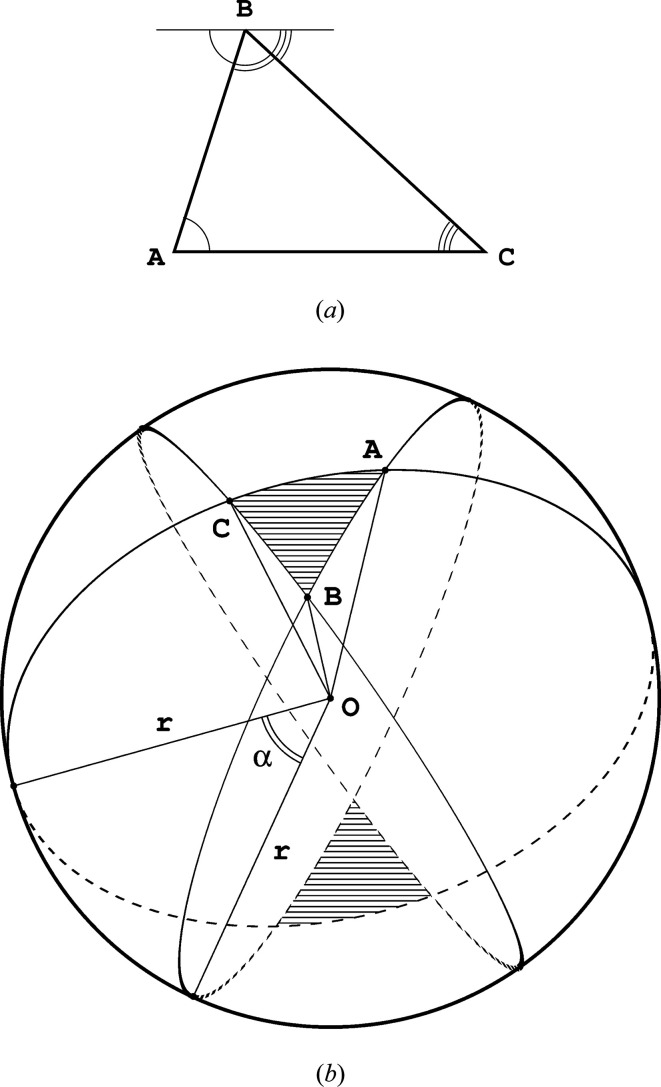
Planar (*a*) and spherical (*b*) triangle *ABC*.

One of the fundamental concepts in geometry is the notion of an angle between two lines. In higher dimensions this generalizes to an angle between planes, hyperplanes *etc*. In various spaces the ensemble of angles in a certain polyhedron satisfies a list of restrictions. A particular relation holds in the planar triangle 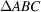 . Its internal angles 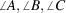 satisfy the fundamental equality [Fig. 2 ▸(*a*)]

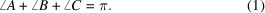




One can generalize the statement above to the sphere. For this purpose, we consider geodesic arcs, *i.e.* arcs of the great circles, which have their center in the center of the sphere and radius equal to that of the sphere. Between three points (vertices) on a sphere that do not belong to a common arc, we can form three geodesic arcs (edges) which bound a region that we call a spherical triangle. In 1603 Thomas Harriot (see Appendix *A* [App appa]) proved that a spherical triangle on the surface of a sphere satisfies a more general equality

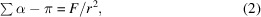

where 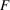 is the area of the spherical triangle, 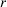 is the radius of the sphere onto which it is inscribed and the sum of the three spherical angles is 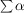 . The proof of Harriot’s theorem is quite elementary and based on the concept of ‘lunes’ (Todhunter, 1886 ▸, pp. 72–73). In essence, every two great circles on a sphere that are not identical dissect the sphere into four regions or lunes [Fig. 2 ▸(*b*)]. Pairwise opposite lunes have the same area *F*. It follows that 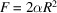 , where 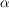 is the opening angle between the lunes. For a spherical triangle with vertices *ABC*, we consider the three pairs of lunes which are generated along pairs of arcs between vertices. Adding up the areas provides the formula given above. For a detailed version of the proof, see Hopf (1940 ▸).

**Figure 3:**
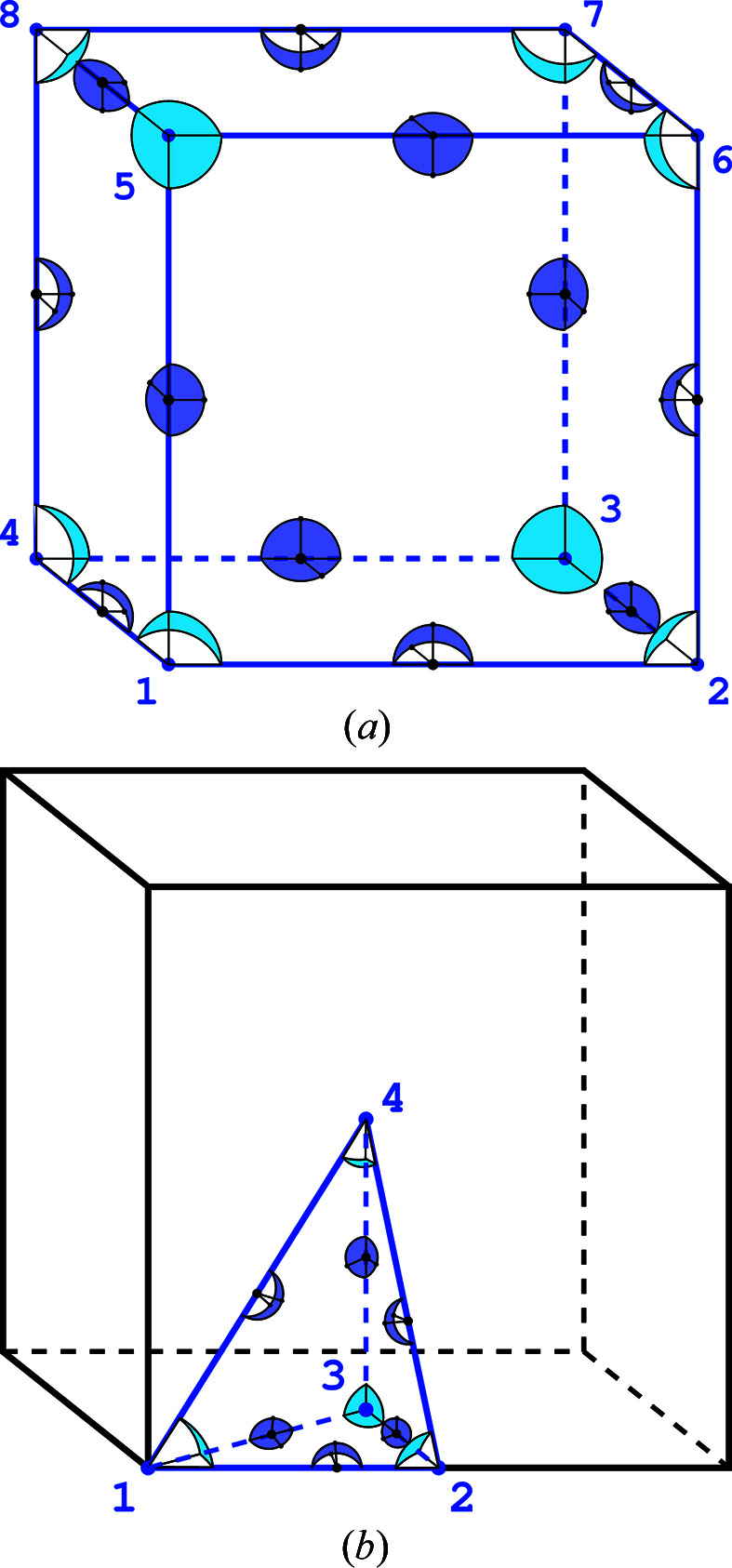
(*a*) In space group *P*1 the ASU covers the whole unit cell and has the shape of a parallelepiped or, as in this special case, a cube. The angles marked in light blue are denoted in the text as 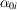 and the angles marked in dark blue as 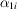 . (*b*) The ASU (blue tetrahedron) embedded within the unit cell (black) of the cubic space group 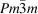 . The internal angles at the vertices (light blue) and edges (dark blue) of the ASU are marked.

The formula for a planar triangle as well as the formula of Harriot for a spherical triangle generalize to higher-dimensional analogs of these figures. In three dimensions one has to take into consideration both the vertex angles and edge angles (Fig. 3 ▸). J.-P. de Gua de Malves (1783 ▸) gave the following formula:

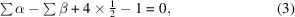

where the first summation goes over all four triplanar angles 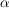 at the four vertices of the tetrahedron, and the second summation is over biplanar angles 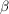 at the six edges between all pairs of faces of the tetrahedron, as marked in Fig. 3 ▸. The contribution 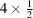 corresponds to the four faces of the tetrahedron with their half spherical angles and the final 1 is the full angle corresponding to the interior of the tetrahedron. Notice the unusual convention: the values of 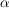 range between 0 and 1 and correspond to the fraction of the area of the unit sphere that the angle subtends inside the tetrahedron. For 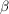 we measure between 0 and 1 the fraction of the area of the unit sphere, centered at any point within the edge, that is cut out by the two planes. In this interpretation the formula obtains a symmetric form,

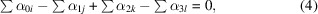

where the summation goes over the angles associated with vertices ( 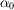 – 0-dimensional elements), edges ( 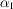 – one-dimensional elements), faces ( 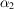 – two-dimensional elements) and interiors ( 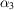 – three-dimensional elements). In higher dimensions this ‘telescoping’ form of the sum remains valid for higher-dimensional ‘triangles’, which are called simplices. The formulas of Harriot and de Gua de Malves were generalized in the theorem of Gram. A complete account of this story is provided by Grünbaum (2003 ▸, ch. 14).

### 2.1. Gram’s theorem

For every *N*-dimensional convex polytope *P* the angle sums satisfy

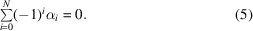

In this formula we always have 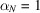 and 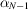 equals half of the number of 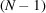 -dimensional faces. In general, 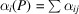 is the sum of all angle contributions from *i*-dimensional elements of *P*.

To clarify this statement let us discuss in detail two examples, based on the ASUs of space groups *P*1 and 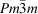 .

In the space group *P*1 the ASU encompasses the whole unit cell, even if accidentally the cell has equal edge lengths and angles, effectively having the shape of a rhombohedron or cube, as illustrated in Fig. 3 ▸(*a*). Each of the eight vertices of the cube contributes 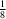 of the whole spatial angle to the interior of the polyhedron, 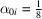 . All 12 edges contribute a quarter of the surrounding space into the cube interior, 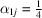 . Again, one-half of the space divided by each of the six faces lies inside the cube, 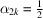 , and there is only one full interior of this solid, 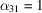 . Thus, we have

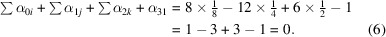




There is only one possible choice of the ASU in the cubic space group 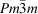 , as a tetrahedron illustrated in Fig. 3 ▸(*b*). All bounding elements of this tetrahedron lie at the special symmetric positions of this space group. Vertices 1 and 4 are positioned at sites of 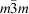 symmetry and transform onto themselves 48 times, and vertices 2 and 3 lie at 16-fold positions with 4/*mmm* symmetry. The edge 1–4 lies along a direction of 3*m* symmetry and sixfold multiplicity, the edges 1–2 and 3–4 lie along directions of 4*mm* symmetry and eightfold multiplicity, and the remaining edges 1–3, 2–3, 2–4 lie along directions of *mm*2 symmetry and fourfold multiplicity. All four faces are positioned at mirror planes and the interior of the tetrahedron lies obviously at a general position of this space group. The fractions of the contributing elements (*k*-cells) residing within the bounds of this ASU are, therefore, as follows:

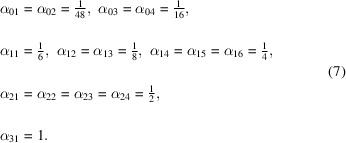




The signed sum of all contributors to the de Gua de Malves formula is therefore

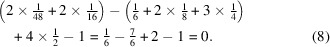




### 2.2. Angular defects

Starting from two-dimensional polytopes embedded in the three-dimensional space, one can talk about the angular defect of a given vertex. This concept is in principle related to a discrete form of curvature (more on this in Section 5 about the Gauss–Bonnet theorem). An angular defect at a vertex is 1 minus the sum of the angles of the faces at that vertex. Here again we use the convention that the angles are normalized with respect to the measure of the given *N*-dimensional unit sphere. For *N* = 1 this measure is 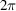 , for *N* = 2 it equals 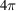 , and in general it is equal to the total surface of the *N*-dimensional sphere.

For example, at each vertex of the cube, all angles between the three pairs of faces meeting at each corner are equal to 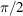 , *i.e.* 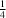 of the full planar angle 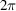 . The angular defect at each cubic vertex is therefore 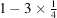 = 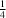 . The sum of the angular defects at all eight vertices adds up to 2.

René Descartes (Appendix *A* [App appa]) discovered [see the historical account by Federico (1982 ▸)] that the total sum of defects 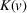 at all vertices 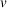 of the boundary 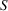 of a 3-polytope 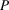 always satisfies

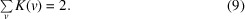

We note here that the value of 2 is equal to the Euler characteristic 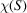 of the boundary of the polytope 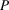 , as fully explained in the next section. In (9) it is crucial that the boundary of the polytope 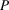 is topologically equivalent to a sphere [otherwise the value 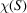 might change]. The notion of the Euler characteristic was of course not known to Descartes; his is, therefore, the true precursor of Euler’s discovery.

According to Hilton & Pedersen (1989 ▸) this formula is equivalent to the original Euler formula 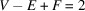 . On the other hand, Descartes’ theorem is a special case of the more general Gauss–Bonnet formula. We will introduce this formula after the discussion of the Euler characteristic.

Descartes’ theorem has an interesting history. According to Grünbaum & Shephard (1991 ▸), ‘The elementary and beautiful theorem known as Descartes’ Theorem was discovered in the seventeenth century and is stated in Descartes’ *De Solidorum Elementis*. The manuscript was lost, however, and we only know of its contents because a copy made by Leibnitz was discovered in the Royal Library of Hanover in 1860. A transcription and translation of this manuscript, together with comments, can be found in Federico’s (1982 ▸) fascinating account of the work.’

In higher dimensions, Grünbaum & Shephard (1991 ▸) found a generalization of Descartes’ formula which still preserves the equality with the Euler characteristic. In their formulation they use a telescoping sum of all defects 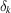 of *k*-cells in an (*N* − 1)-polytope 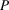 embedded into 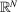 in which an intersection of any two (*N* − 1)-faces is either empty or an (*N* − 2)-face, forming altogether a polytope which is a topological manifold. The formula is

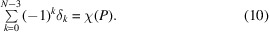




Note that 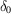 is the sum of defects at all vertices (0-cells), 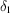 is the sum of all defects at all edges (1-cells) *etc*. A nice example is an empty hypercube in dimension 4 (with cells up to dimension 3). It is a union of eight three-dimensional cubes, where four 3-cubes meet at each of the 16 vertices. The defect at each vertex is 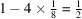 since the solid angle at a vertex of a 3-cube is 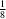 . Therefore, the defect 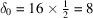 . The hypercube has 32 edges, with three 3-cubes meeting at each edge. Hence the edge defect equals 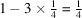 since the angle between two faces of the 3-cube is 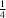 . In total, we have 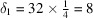 . We conclude, therefore, that the Euler characteristic of the empty hypercube (without the internal 4-cell) equals 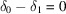 . This agrees with a general statement from topology that a 3-manifold has the Euler characteristic equal to zero.

**Take-home message**

The total angular defect of a polytope is a quantity that, despite its very geometric origin, is a topological invariant.

## 3. The Euler characteristic

One of the most fundamental ideas in mathematics is the notion of ‘counting of objects’. In its simplest form, given two sets 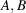 , one can attach to them their cardinality (Appendix *B* [App appb]) (which in the case of finite sets is simply the number of elements), denoted 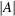 and 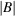 , respectively. For two finite sets the most fundamental property of the counting function can be encoded in two statements:

(i) The size of a set {*} containing only one (any) element equals 1, *i.e.* the cardinality |{*}| = 1.

(ii) The counting function 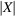 of a set 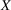 is compatible with operations on finite sets 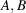 (*i.e.* with sum 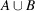 and intersection 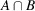 ):

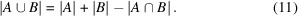




Property (ii) indicates that the counting measure 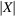 of a finite set *X* is a valuation (Appendix *B* [App appb]). In general, a valuation *v* on a collection *S* of sets is a function from *S* to the set of real numbers such that

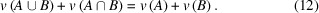




In its general form, property (ii) is called the inclusion–exclusion principle:

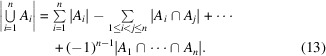




When the sets that we encounter are infinite, the cardinality of a set lacks the natural valuation property. In particular, when dealing with (compact) (see Appendix *B* [App appb]) polytopes in the Euclidean space 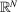 (which contain infinitely many points), the cardinality of a polytope would not constitute a sensible valuation – we need something much finer.

We denote by 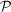 the set of polytopes (of varying dimensions) that can be obtained from the finite unions and intersections of convex closed polytopes.

*Valuative definition of the Euler characteristic*. The celebrated theorem of Hadwiger (Klain & Rota, 1997 ▸, Theorem 5.2.1) states that there exists a unique function 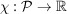 such that:

(i) 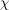 is invariant under rigid motions of the polytope 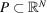 , *i.e.* 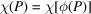 where 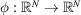 is either a rotation or a translation.

(ii) 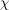 is convex continuous, *i.e.* for a sequence of convex polytopes 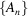 of fixed dimension which approach a convex polytope *A* with respect to the Hausdorff metric (Appendix *B* [App appb]), the value 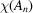 approaches 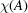 in the limit 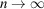 .

(iii) 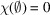 for the empty set 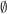 . For any 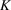 which is a non-empty convex compact polytope (of arbitrary dimension), we have 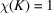 .

(iv) 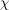 is a valuation, *i.e.* for any 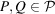 we have

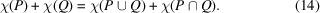




**Figure 4:**
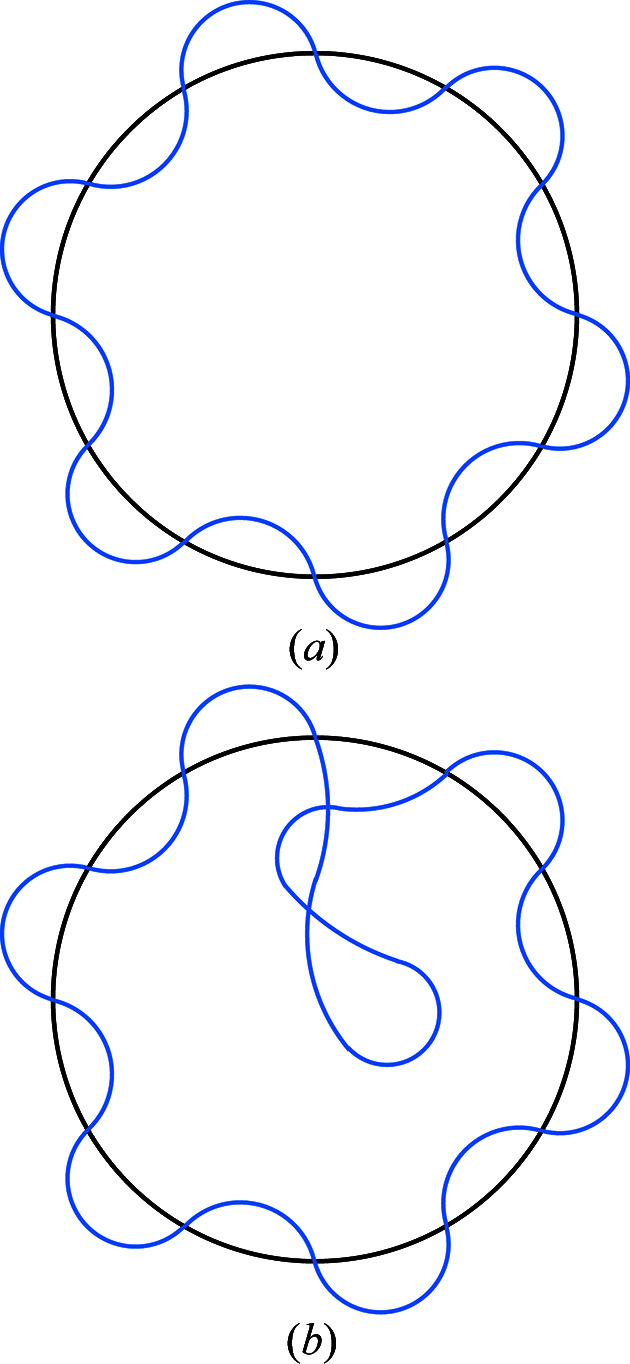
The Hausdorff distance between shapes in panel (*a*) is smaller than the distance between shapes in panel (*b*). In the latter case the distance increases because the necessary fattening must be larger to encompass the intertwining inner loop.

Remarks:

(*a*) This definition is powerful enough to let us compute the value of 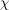 for any polytope in 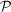 . For historical reasons the number 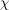 is called the Euler characteristic (Appendix *B* [App appb]).

(*b*) The statements (i)–(iv) above are referred to as ‘properties’ of the function 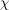 .

(*c*) The normalization of 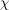 in property (iii) makes the condition (ii) rather trivial. However, the properties (i), (ii) and (iv) [without (iii)] determine other convex-continuous valuations (like volume integrals, surface integral *etc*.).

(*d*) The Hausdorff metric used in property (iii) allows one to generalize the usual Euclidean distance between points to collections of multiple points (such as polytopes). We present in Fig. 4 ▸ two pairs of polytopes – two that are close in the sense of the Hausdorff metric [Fig. 4 ▸(*a*)], and two that are ‘far apart’ [Fig. 4 ▸(*b*)]. Continuity of a given function *f* with respect to the Hausdorff metric means that a small change in the value of the Hausdorff distance of two arguments (polytopes) implies a small change in the value of the function *f* against these arguments.

Properties (i)–(iv) allow us to design a game, ‘Let’s compute Euler’s number’ in Section 3.2. The rules are rather simple. We start from a given shape, which is our challenge. In each step all we can do is to decompose the shape into two parts for which we try to compute the Euler characteristic separately. Then we add these numbers and compute the Euler characteristic for all intersections of the pieces and apply the rules of alternating sum. There are two tricky aspects of the game: if the shape is polytopal, the game will always end (think about triangulation of the space); the result (value of the Euler characteristic) does not depend on the way we play a particular round. This surprising conclusion can be proven on the basis of either the combinatorial or topological formula for the Euler characteristic.

*Combinatorial definition of the Euler characteristic*. Euler, Schläfli and Poincaré defined, at various levels of generality, the Euler characteristic of a polyhedral complex 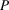 as

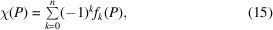

where 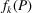 denotes the number of *k*-cells of *P*. It is possible to check that the function defined in such a way satisfies principles (i)–(iv).

In the most classical form, for a polyhedral surface 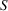 (*e.g.* a boundary surface, or ‘skin’, of a solid convex 3-polytope in 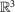 ), if 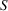 has 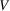 vertices, 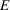 edges and 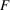 faces we have

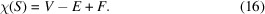




In particular, for such a polyhedral skin *S* of a solid convex 3-polytope the celebrated Euler theorem is (Euler, 1758 ▸)

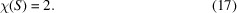




In his wonderful book Richeson (2008 ▸) explores the many facets of this remarkable formula.

In fact, formula (15) can be deduced from the properties (i)–(iv) of 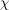 as was shown by Klain & Rota (1997 ▸, Theorem 3.2.3). This means that the valuative definition of 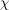 provided by Hadwiger, while being rather modern compared with the definitions of Euler, Schläfli and Poincaré, is a much more natural point of departure for our discussion. Such an achronological state of affairs is not uncommon in mathematics.

*Topological definition of the Euler characteristic*. The Euler characteristic can be extended to any topological space *X*. The meaning of 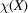 in such a case is the alternating sum

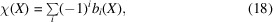

where 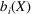 denotes the *i*th Betti number of *X* and the summation index runs from 0 to the dimension of the space *X*. An *i*th Betti number of *X* is the number of *i*-dimensional ‘holes’ in *X* (Richeson, 2008 ▸, ch. 23; see also Hatcher, 2002 ▸; Spanier, 1982 ▸). The dimension of the ‘hole’ is related to its boundary, rather than its interior (a sphere bounds a ball-like hole *etc*.). A circle 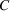 bounds a disc-shaped hole, hence 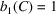 . A sphere *S* bounds a ball but it has no disc-like hole, hence 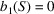 , 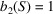 . In the familiar context when *X* is a polytope *P*, the combinatorial and topological definitions of the Euler characteristic coincide. While the terms of the telescoping sums (15) and (18) are rather similar in appearance, they are not directly comparable. The Betti numbers 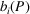 of a polytope 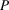 cannot in general be deduced from the number of faces 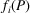 . For example, the Betti numbers of a polytopal skin of a convex polytope are always 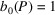 , 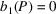 , 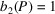 , 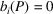 , 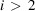 , while the numbers 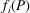 will vary with each polytope 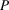 .

However, we note here that the valuative, combinatorial and topological definitions of the Euler characteristic do coincide for polytopes. That means we should recognize the quantity 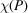 as something more fundamental than any of these three definitions.

### 3.1. What is homotopy equivalence and how is it related to the Euler characteristic?

Homotopy equivalence (Appendix *B* [App appb]) is a notion that was discovered during the formative years of mathematical topology. The main idea of homotopy equivalence is to be able to ‘bend one space into another’. In essence, homotopy equivalence between two spaces *X* and *Y* preserves the essential topological features, such as path connectivity and the number of connected components.

This ‘bending’ is done with respect to very strict assumptions: the transformation that replaces one space by another is continuous, which intuitively means that we deform spaces without tearing them apart. So, we say that a subset of the Euclidean space 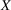 is homotopy equivalent to a subset 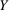 if there exist two maps 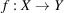 and 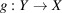 , both continuous and such that their compositions 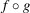 and 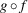 are homotopic (Appendix *B* [App appb]) to the identity maps (an identity map is just sending an element to itself) on 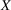 and 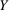 , respectively.

**Figure 5:**
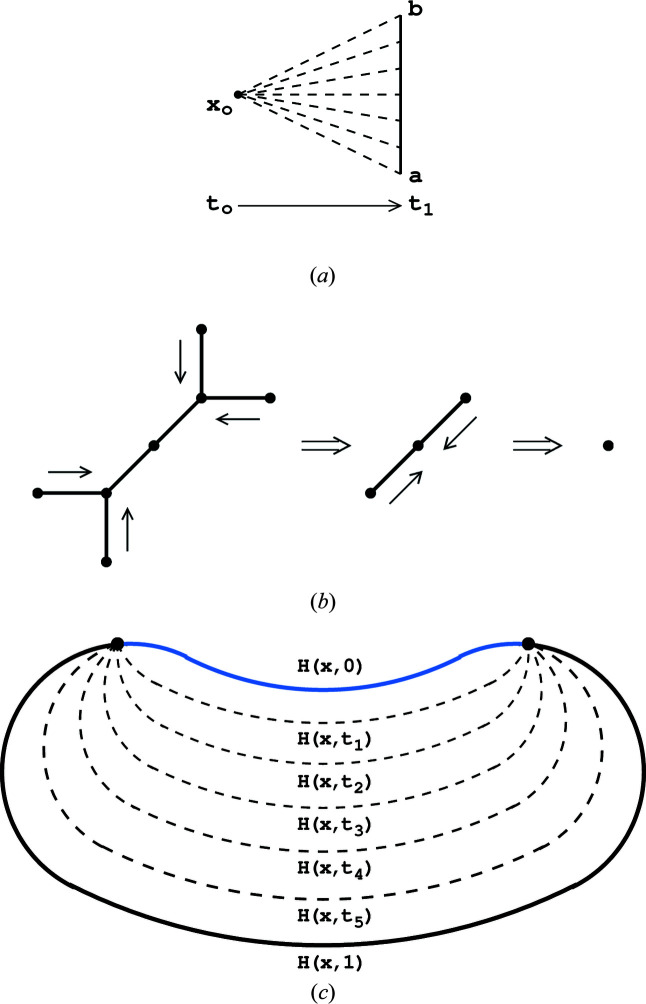
(*a*) An illustration of the process of transforming a single point 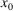 of the closed interval 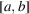 into the interval itself. The transformation is a homotopy map 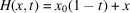 . (*b*) An illustration of how a homotopy can deform in two steps a six-segment edge graph into a single point. In the first step we contract the four outer segments into their end points using the homotopy from Fig. 4 ▸(*a*). Next, we contract with a similar homotopy the remaining two segments to the central point. (*c*) An example of a homotopy process 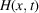 , in which a curve with two fixed end points is continuously transformed into another curve. The intermediate steps of the evolution in time are denoted with 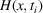 for time points 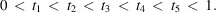

In particular, a space is homotopy equivalent to a point only if there exists a point within this space such that every other point is connected to it by a connected path (but sometimes this is not enough – see the example of the circle and a point at the end of Section 3.2). For example, any star-shaped polytope is homotopy equivalent to a point, and so is any convex subset. The necessary homotopy (Appendix *B* [App appb]) is realized by moving each point onto a fixed point along a line which is contained within the space. In Fig. 5 ▸(*a*) we visualize how a point is homotopy equivalent to a bounded segment. The homotopy moves linearly each point of the segment onto one particular point of the segment. Similarly, in Fig. 5 ▸(*b*) we show how a polygon which consists of several connected segments is homotopy equivalent to a point. The homotopy ‘shrinks’ the branches in several stages. Finally in Fig. 5 ▸(*c*) we demonstrate how two paths on the plane with the same start and end points transform under homotopy into each other in a finite time 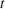 .

For two polytopes (not necessarily convex or connected) the homotopy equivalence between them will save the general connectivity between points but might even lower the dimension. Take a square with a smaller open square removed and a one-dimensional rectangular skeleton (a parallelogram) – they are homotopy equivalent, but definitely not rigid-motion equivalent. However, the Euler characteristic remains the same.

With the topological extension of 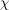 we gain the extra flexibility of the homotopy invariance, 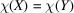 if 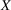 is homotopy equivalent to 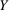 . The topological extension of the definition of the Euler characteristic also satisfies the usual principles (i)–(iv). In the first rule we can replace the rigid motion with any homotopy equivalence, and in principle (iii) the convex polytope can be replaced with any topological space that is homotopy equivalent to a point.

### 3.2. ‘Let’s compute Euler’s number’ game

We are now ready to explain explicitly the computation of the Euler characteristic for several spaces. We encode the principles (i)–(iv) into the following rules:

Rule A: the Euler characteristic of any two homotopy-equivalent spaces is the same. This rule also includes all rigid motions of polytopes.

Rule B0: the Euler characteristic of the empty set equals 0.

Rule B1: the Euler characteristic of a space homotopy equivalent to a point equals 1.

Rule C: for any two spaces 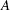 and 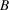 we have the equality

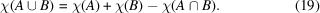




These rules are now used to define a game that we call ‘Let’s compute Euler’s number’. The word ‘game’ means here an engaging classroom activity. Below are four example runs of the game.

#### 3.2.1. Game 1

**Figure 6:**
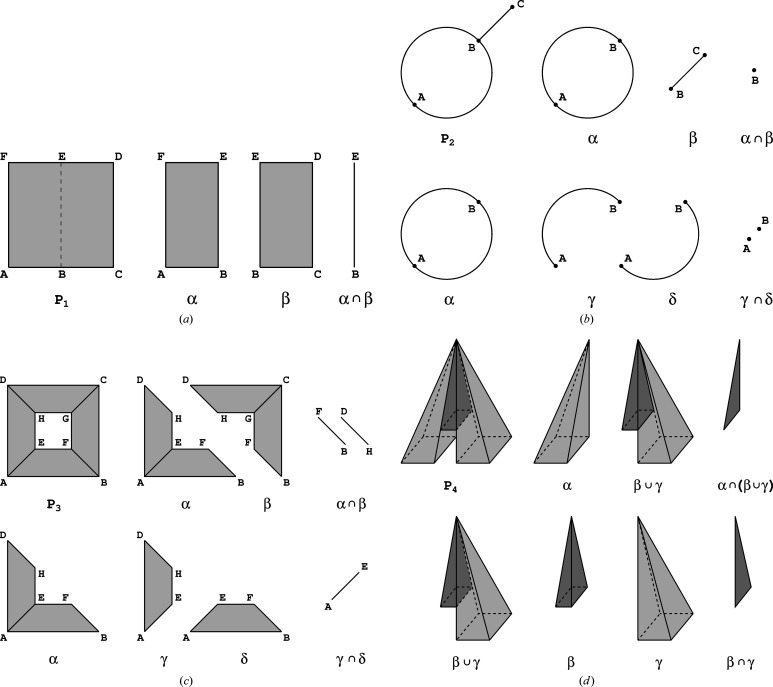
Panels (*a*)–(*d*) provide graphical illustrations of the four games 1–4 described in detail in the text.

We start with a filled square on the plane [Fig. 6 ▸(*a*)]. According to rule B1, since the filled square is convex, its Euler characteristic equals 1. Alternatively, we argue that there exists a linear motion, continuous from point to point, which moves each point of the filled square onto its center.

In an alternative (not optimal) run, we can dissect the filled square 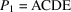 into a union of two ‘halves’, which are filled rectangles 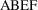 and 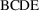 , intersecting along one edge 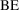 . Let us call these rectangles 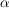 and 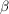 . The Euler characteristic of each piece equals 1 due to rule B1 and the Euler characteristic of 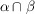 is 1 as well (an edge is also convex). Hence 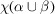 = 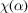 + 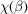 − 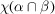 = 1 + 1 − 1 = 1. As a result, we can see that the decomposition did not affect the final result.

#### 3.2.2. Game 2

Let us consider a shape 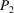 that consists of a circle 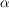 and an edge 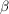 attached to this circle at one point 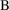 [Fig. 6 ▸(*b*)]. The shape 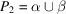 is an empty lollipop. Rule C tells us that

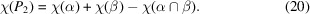




Rule B1 informs us that 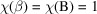 , hence 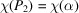 . For a trained topologist this is not surprising since every such ‘hairy’ circle is homotopy equivalent to a pure circle. So how do we compute 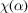 ? Well, a circle is a union of two closed half-circles 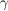 and 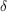 which intersect at the union of two points 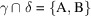 . Therefore, we have

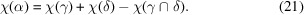




Rule B1 tells us that 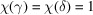 (now we use the topological version of this rule) and the application of rule C gives

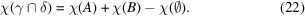




Finally, we obtain 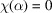 and 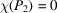 .

#### 3.2.3. Game 3

Let us consider a rectangle with a smaller rectangle removed from its interior [Fig. 6 ▸(*c*)]. The position of the interior rectangle (as it will turn out) is not important. What is important is that we do not remove the inner boundary around the hole. From a topological point of view, this space is homotopy equivalent to an ordinary circle (in a sense, the flexibility of topology ruins the excitement of our game…). So, the conclusion should be 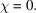


In the polygonal version, we can decompose the shape 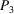 into a union of four trapezoids 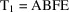 , 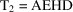 , 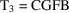 , 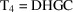 , with parallel sides corresponding to one outer and one inner edge of the hollow rectangle. Each trapezoid 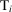 satisfies 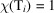 since it is a convex shape. Pairwise neighboring trapezoids, say 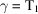 and 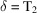 , have an edge 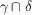 as their intersection, hence 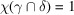 , according to rule B1. Let 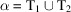 and 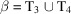 . It follows from rule C that 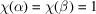 . The same rule implies that

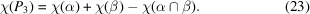




The intersection 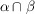 is a union of two disjoint edges 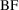 and 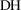 . It follows from rule C that 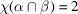 and finally 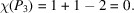


#### 3.2.4. Game 4

Finally, let us try a three-dimensional case. Fig. 6 ▸(*d*) shows a square pyramid with a tunnel cut out at its bottom. We cut the original shape 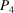 into three convex pyramids 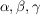 , two of them ( 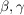 ) connected along a face ( 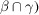 . It follows from rule C that the Euler characteristic of the union 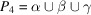 satisfies 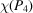 = 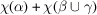 − 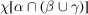 .

Rule B1 implies that 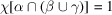 . Next, rule C implies that

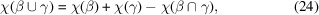

and 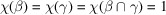 by rule B1. In total, we calculate that

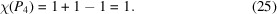




#### 3.2.5. Further challenges

Now, our readers, equipped with such a powerful tool, are asked to try to compute the Euler characteristic of the following shapes:

(1) A rectangle with two holes (of any shape). (Answer 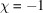 .)

(2) A torus surface (hint: cut the torus surface vertically into two bended tubes; argue that the Euler characteristic of such a tube is the same as for a circle; the intersection of these tubes is a union of two disjointed circles). (Answer 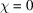 .)

(3) A ball. (Answer 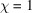 .)

(4) A sphere. (Answer 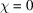 .)

(5) A union of two filled rectangles that meet at a single corner, and which are glued to a filled triangle that is attached by its side to the corresponding edge of one of the rectangles. (Answer 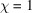 .)

(6) A square pyramid as in Game 4, but with two perpendicular tunnels drilled at its bottom, running parallel to the base edges. (Answer 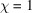 .)

(7) A cube with a tunnel drilled through its center. (Answer 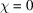 .)

(8) A cube with two crossing tunnels drilled through its center. (Answer 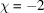 .)

(9) A cube with three crossing tunnels drilled through its center. (Answer 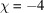 .)

(10) Challenge: can you generalize the formula for a cube drilled with *n* tunnels meeting at one central point? [Answer: 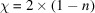 .]

We note that, in cases where for two spaces 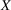 and 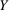 the Euler characteristic is different, these spaces are not homotopy equivalent. However, even when 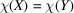 , it does not imply in general that such spaces are equivalent. A simple and fun proof (without any algebraic topology) that a circle is not equivalent to a point is provided by Brown (1974 ▸).

**Take-home message**

The computation of the Euler characteristic of a polytope (or even of topological space) is a valuation process which measures the essential ‘connectivity’ properties of a given set. It is a natural, yet non-trivial, extension of the counting measure for finite sets, which remains finite for polyhedra.

## 4. The modified Euler characteristic

A typical object in crystallography is a polytope, such as the unit cell or its ASU, which is propagated through space by the action of a certain space-group symmetry. Such a construction provides a new point of view on the intrinsic geometry of the polytopes. Instead of studying a single solid polytope in space, for which the Euler characteristic equals 1, we need to take into account the possibility of vertices, edges and faces being shared by adjacent polytopes in the tessellation.

To fix that, one has to introduce a new principle of sharing. In this new ‘modified Euler characteristic’ ( 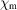 ) concept we count the *k*-cells in the minimal space element (in crystallography, the ASU or Dirichlet domain) which is replicated under the space-group action, with the contribution of each *k*-cell normalized by a weight that is inversely proportional to the number of sharing neighbors, or by the fraction of the total solid angle around this *k*-cell subtended by the polytope in question. A quantity that will abstract from the particularities of the tessellation subdivision is termed the orbifold Euler characteristic (Appendix *B* [App appb]), as introduced by Satake (1956 ▸) and Thurston (2002 ▸). We have investigated the concept of modified Euler characteristic in earlier papers (Dauter & Jaskolski, 2020 ▸; Naskręcki *et al.*, 2021*a* ▸) and in relation to the orbifold notion as well (Naskręcki *et al.*, 2021*b* ▸). The modified Euler characteristic has the form

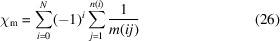

where 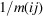 is the fraction the individual element *j* of dimensionality *i* contributes to one selected polytope. In three-dimensional space this can also be expressed as

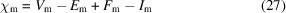

where 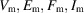 represent the total fractions of elements of different dimensionality ascribed to one polyhedron in the three-dimensional lattice (Dauter & Jaskolski, 2020 ▸; Naskręcki *et al.*, 2021*a* ▸).

The modified Euler characteristic is multiplicative with respect to coverings of the spaces. The tessellation by its periodic behavior corresponds to a space named the *N*-dimensional torus. Every torus is obtained by ‘gluing’ the appropriate cells. In particular, in dimension 2 we start from a filled square. We glue pairwise in the same orientation the two vertical edges, as well as the horizontal edges. In effect, this new space allows one to ‘pass through the wall’. The effect of crossing the northern boundary is to return to the southern border. A similar situation arises when crossing east to west. In three dimensions we identify and glue together the corresponding faces of the cube, to obtain a three-dimensional torus. In general, one can imagine a filled hypercube in dimension *N*, where we perform a compatible identification of the (*N* − 1)-cubes.

Such a torus maps onto a space which is an orbifold. This object looks locally like a polytope (or even an ASU) except for some special points at which the neighborhoods are rather unusual (Naskręcki *et al.*, 2021*b* ▸).

**Take-home message**

The fundamental principle that emerges from the calculations of 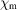 is that for all space groups the modified Euler characteristic of the ASU equals 0.

Therefore, the modified Euler characteristic of a periodic tessellation is a useful invariant of the tessellation. A practical computation of the modified Euler characteristic can be performed in a way that resembles the original Euler game. Each time we divide a *k*-cell along a (*k* − 1)-cell, the latter inherits the weight of the former.

Below we present two examples of the computation of the modified Euler characteristic.

**Figure 7:**
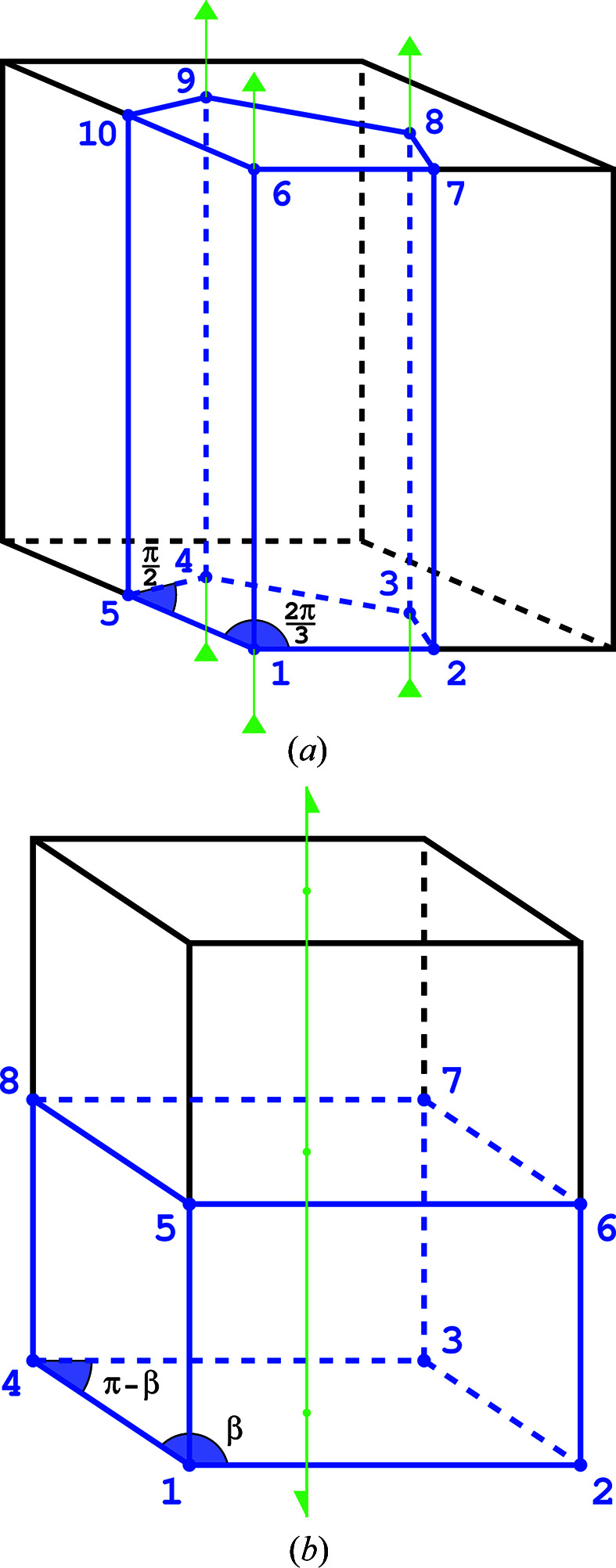
(*a*) In space group *P*3 the ASU (marked in blue) covers 1/3 of the unit cell (black). The representative planar angles within the horizontal face of the ASU are marked. (*b*) In space group *P*2_1_ the ASU corresponds to one-half of the unit cell. The planar angle at four vertices is the monoclinic angle 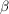 and at the four other vertices it is 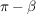 .

*Space group P3 example*. In space group *P*3 the ASU recommended in *International Tables for Crystallography*, Volume A (Aroyo, 2016 ▸), is a prism with a pentagonal base [Fig. 7 ▸(*a*)]. Each of the three lower and three upper vertices lying at the threefold axes provides 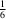 of the total spatial angle inside the ASU, and the four remaining vertices provide 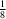 each of the total angle. The 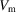 value is therefore 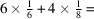 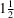 . Each of the three vertical edges positioned along the threefold axes provides 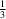 of the total angle and the remaining 12 edges give 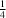 of that angle each. The total value is therefore 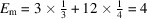 . The seven faces give in total 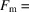 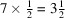 . With one full interior we get

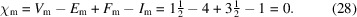




*Space group P2_1_ example*. In space group *P*2_1_ the ASU encompasses the lower half ( 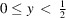 ) of the unit cell [Fig. 7 ▸(*b*)]. Every second horizontal planar angle at the ASU vertices has the monoclinic value of 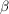 and the remaining vertices have the complementary angle 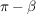 . In effect, the average value of all these angles is 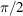 , and the total internal spatial angle of all eight vertices is 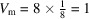 (in analogy to the cube). All edges contribute in total 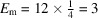 . The contribution of the six faces is 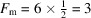 . The resulting value is

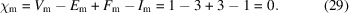

One can further note that the formula of de Gua de Malves is a special form of the modified Euler characteristic, which always takes the value of 0. When we replicate a given polyhedron through space, the vertices, edges and faces are appropriately shared among three-dimensional cells. The weight with respect to which each *k*-cell is counted in the modified Euler characteristic formula corresponds to the fraction of the total angle that the given *k*-cell subtends from the full solid angle. That means that, in the context of polytopes that are replicated by space-group action, we can reinterpret the modified Euler characteristic in terms of angles rather than weighted counts of shared *k*-cells. The examples in Figs. 3 ▸(*a*) and 3 ▸(*b*) perfectly illustrate this point.

## 5. Gauss–Bonnet formula

The formula of Harriot and its higher-dimensional analogs encode what modern mathematics calls the curvature around a point. In simple terms, a curvature (Gauss curvature) measures how the space ‘bends’ around a single point and quantifies it as a real number (negative, zero or positive). We can have positive curvature, *e.g.* of a point on a sphere. Zero curvature means that the space around a point is flat. For negative curvature things are somewhat upside down and spaces like that for a human standing at a particular point would look unnatural in the sense of revealing more of the horizon than expected. A saddle point is a good example of a place with negative Gaussian curvature (Richeson, 2008 ▸, ch. 21).

The measure of the curvature on a surface *S* can be used to express the total value of such a deformation. The Gauss–Bonnet theorem states that for a closed surface the total measure of the Gaussian curvature equals 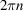 for a certain integer *n*. In precise terms

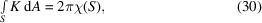

where the integer 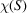 is the Euler characteristic of *S*. The integration goes over the surface *S* with respect to the surface measure 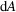 (Richeson, 2008 ▸, ch. 21).

Let us investigate a simple example. For a sphere of radius *R* its Gaussian curvature *K* is constantly equal to 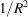 at any point of the sphere. Hence the integral

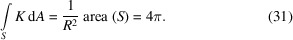




This leads us to the conclusion that 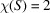 for the surface of a sphere, independently of its radius, a conclusion we can also explain using the combinatorial properties of the Euler characteristic. This is in fact remarkable, because for other, more wobbly closed surfaces that are distortions of a sphere and can be treated as homotopy equivalent to it, the Gaussian curvature *K* will obviously change locally, leading to an extremely complicated integration problem. Yet, the final result is always the same, 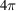 in three-dimensional space.

**Take-home message**

The total Gaussian curvature of a closed surface is a quantity that is independent of any small (*i.e.* continuous) deformations of this object. Its nature is purely topological.

In this paper we are mostly interested in the curvatures, angles, topological properties *etc*. of polyhedra and polyhedral tessellations of spaces. In particular, we need to develop and discuss the notion of the curvature for a space with edges and boundaries.

For a surface built out of two-dimensional polygons, the faces are flat, and hence their Gaussian curvature is 0. The bending of the space is concentrated on the vertices. Specifically,

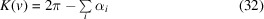

is the angular defect at a vertex 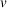 , as in Fig. 2 ▸(*b*). The summation is over face angles adjacent to 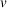 . Summing the defects 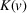 over all vertices 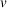 of a polytopal surface we obtain a discrete analog of the Gauss–Bonnet theorem (30):

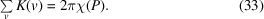




Note that the most classical case of the polyhedron homotopic with a sphere reveals the equivalence of the discrete Gauss–Bonnet theorem with the formula of Descartes [equation (9)]. There are also generalizations of (30) and (33) to spaces with boundaries or of higher dimensions. In the discrete setting, a miracle happens again and the right-hand side of formula (33) equals 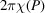 , where 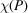 is the Euler characteristic of the polyhedral surface 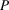 . In simple geometric terms, the number 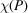 is computed by counting the number of ‘holes’ in the polyhedron 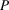 . For example, a polyhedral torus surface has exactly one hole. An empty cube has no holes through its surface *etc*. Eventually, the Euler characteristic 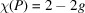 where 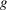 is the number of holes in 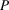 .

## 6. Vistas

We have discussed many interesting connections between the alpha (angle), chi (Euler characteristic) and kappa (curvature) worlds. The key result that connects the three worlds is Descartes’ theorem, which links the total angular defect with the Euler characteristic. While being a result about angles, it is the simplest variant of the Gauss–Bonnet theorem for polytopes.

The formula of Harriot provides a link between the angle sums, Euler characteristic and modified Euler characteristic for tessellations of space. In higher dimensions such a comparison can be made via the theorem of Gram, as explained above.

In our rather elementary considerations throughout this paper, we have not discussed in detail that the modified Euler characteristic can be interpreted as the Euler characteristic of an orbifold space associated with the tessellation. A detailed discussion of this view of our topic is presented by Naskręcki *et al.* (2021*b* ▸). This high-brow point of view makes it possible to prove in an elegant way that the modified Euler characteristic is zero for every tessellation in every Euclidean space, using only the multiplicativity of the Euler characteristic under coverings of spaces and the vanishing of the modified Euler characteristic for a simple cubical tessellation (which corresponds on the orbifold side to a wrapped torus space). The Gauss–Bonnet theorem has its version for (two-dimensional) orbifolds. Yet again, this establishes a connection between counting angles in polytopes shared in the tessellation and the modified Euler characteristic.

Finally, we note that the terms that appear under the sum signs in the modified Euler characteristic may be interpreted as the number of *k*-dimensional cells in the tessellation of space. More precisely, if we are given a certain space tessellation and we fix a vertex of a particular polytope as the center of this tessellation, then, growing with the sphere radius 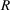 , we obtain a counting function 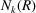 which computes the number of *k*-dimensional cells which are strictly contained in or intersect a ball of radius 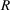 centered at this fixed vertex. Coxeter (Appendix *A* [App appa]) (1948 ▸, ch. 4.8) studied such functions and observed that the leading term coefficients 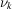 [so that 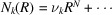 ] satisfy the formula 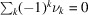 for the periodic tessellations (under certain simplifying conditions). We have explored this theme (Naskręcki *et al.*, 2021*b* ▸), proving that this formula is essentially the modified Euler characteristic of the orbifold associated with the tessellation.

## 7. Conclusions

The notion of the Euler characteristic of a space, polyhedron *etc*. is a well established numerical quantity, known for many years in mathematics. Over the past five decades, due to advances in our understanding of topological and differential aspects of polytopes, several new variants of the Euler characteristic have been proposed. The first formulation of Euler was somewhat ahistorical to the following development and proved to be less fundamental than the combinatorially founded concepts introduced later.

In particular, the modern notion of an orbifold plays a key role in this development, as well as in modern applications driven by computer graphics. Also, a reformulation of the Gauss–Bonnet equation combines the local differential input, expressed in the Descartes angular defect formula, with the global idea of the Euler characteristic of an orbifold space. Such a point of view sheds new light on the intricate relations between combinatorially computed data of polyhedra and tessellations. Our aim in this paper has been to make the crystallographic community aware of these modern notions and indicate their practical and very concrete nature, as well as the unexploited potential for applications in the computational and numerical aspects of crystallography.

In the general context, the many facets of the modified Euler characteristic reveal the unity and beauty of mathematics and crystallography, combining their many flavors: combinatorial, geometric and topological. We hope that interested readers will benefit from this unified exposition and will view the modified Euler characteristic as a versatile tool that allows the qualitative properties of various spaces to be measured.

## Figures and Tables

**Table 1:** Formulas and theorems discussed in the paper

Name of the theorem	Dimension of the object	Dimension of the space
Triangle angular formula	2	2
Harriot theorem	2	3
de Gua de Malves formula	3	3
Gram theorem	*N*	*N*
Descartes’ theorem	2	3
Grünbaum & Shephard theorem	*N* − 1	*N*
Gauss–Bonnet theorem	2	3
Discrete Gauss–Bonnet theorem	2	3
Euler’s formula	2	3
Vanishing of the modified Euler characteristic for crystallographic tessellations	*N*	*N*
Equivalence between different definitions of the Euler characteristic	*K*	*N*

## Appendix A Biographical notes (in alphabetical order)

Pierre Ossian Bonnet (1819–1892) was a prolific mathematician and teacher of the 19th century. In his early life he considered a career as an engineer but he eventually turned to analysis and geometry. He taught at École Polytechnique and the Sorbonne in Paris, working at that time on several ideas in differential geometry. His interests were broad and included cartography, algebra and mathematical physics. In particular, he applied certain ideas from differential geometry to geographical maps, including an extension of Lambert’s ideas about transformations which preserve angles on cartographic maps. His greatest contribution was the extension of the ideas of Gauss about curvature of surfaces and the proof of the Gauss–Bonnet formula for a surface with boundary.

Harold Scott MacDonald Coxeter (1907–2003) was a man of many talents. An accomplished pianist and a talented linguist, he finally chose mathematics as his master subject, much to the benefit of humanity. Educated in Cambridge, he spent some time at Princeton and returned to Trinity College where he was appointed as a lecturer. He became later a professor in Toronto. He made major contributions to the theory of polytopes, the study of reflection groups and tessellations of spaces.

René Descartes (1596–1650) was a French philosopher and mathematician. He served for a while in the French and Dutch armies, but later he mostly lived in the Netherlands. He died from pneumonia in cold Sweden as a tutor of Queen Christina. With his famous maxim *cogito ergo sum*, he is regarded as the precursor of modern rationalistic philosophy, pioneering the ‘Age of Reason’. Descartes left an enduring legacy in mathematics. He introduced the Cartesian system of coordinates and algebraic methods for analyzing geometrical problems. Descartes was a prominent figure of the scientific revolution in the 17th century.

Leonhard Euler (1707–1783) was one of the greatest and most prolific mathematicians of all time. Swiss by birth, he spent most of his life in Berlin and St Petersburg, where he is buried. He influenced many branches of mathematics, including calculus, analysis, number and graph theories, complex function theory, and topology. In crystallography Euler is remembered for his  representation of complex numbers, for his theorem about the number of fivefold axes (12) in solids with icosahedral symmetry, or for his formula relating the number of vertices (  ), edges (  ) and faces (  ) of any solid (  ). His famous identity  is considered the most beautiful formula ever.

Carl Friedrich Gauss (1777–1855) is probably the greatest mathematician of all time, called ‘*Princeps mathematicorum*’; he very strongly influenced many branches of mathematics and science and tutored many famous mathematicians. Gauss was intellectually prodigious at a very young age. He studied at the universities at Braunschweig and Göttingen, where he later lived. His most notable contributions were in number theory, geometry, probability, geodesy and astronomy. He contributed to important research on magnetism and his name is used as a unit of magnetic induction. Gauss left many important unpublished ideas, extending his influence throughout the 19th century.

Thomas Harriot (*circa* 1560–1621), in today’s terms, was a scientist, mathematician and explorer. In 1585–1586 he was part of the British Crown’s expedition to the New World (called Virginia) to assess the economic value of the new colonies. Tasked with finding the best way of stacking cannonballs on ship decks, he discovered, without publication, ‘Kepler’s conjecture’ approximately 20 years before Kepler, *i.e.* around 1591. Most of Harriot’s discoveries and inventions are documented only in his sparse notes. The only published opus is on the exploration of the New World colony.

Henri Poincaré (1854–1912) was educated at École Polytechnique in Paris and remains one of the very few mathematicians who understood the field in all its aspects. He made seminal contributions throughout mathematics, competing with Einstein in the discovery of the principles of general relativity. His foundational work in topology transformed the field completely, leading to further development of algebraic topology and making it possible to provide a topological definition of the Euler characteristic. He actively developed mathematical physics, in particular contributing to the qualitative understanding of the solutions of differential equations.

## Appendix B Glossary of terms (in alphabetical order)

Angular defect. A measure of curvature of a polytope at a given vertex. It can be expressed as the angle needed to complement the sum of the angles meeting at a given vertex to the full angle.

Cardinality. The number of elements in a set.

*k*-Cell. A *k*-dimensional building block of a polytope.

Compact set. In the Euclidean space  a set is compact if and only if it is a closed and bounded subset of the space.

Continuous function. A function *f* is continuous if for any *a* in the domain of *f*, if *x* is close to *a*, then  is close to  . The closeness of points is measured by a precise condition which depends on the topology of the domain and codomain.

Covering. A covering map  between two topological spaces is a continuous map such that for every point  its preimage set  consists of points *x* such that some neighborhood  of *x* is homeo­morphic with a suitable neighborhood of *y*. A covering is finite if every preimage set is finite (and in fact the number of elements in each preimage is the same number  ). The number  is called the degree of the covering.

Curvature. A number that measures the flatness of space at a given point.

Hausdorff metric. Given two compact sets  in Euclidean space, we can construct for each of them an ɛ-fattening  , which is a set of elements of space which are within the distance ɛ from some point in *X* and *Y*, respectively. A Hausdorff metric is a function  that satisfies the property

If it is not possible to achieve one of the inclusions for any ɛ, then the distance  is declared to be infinite.

Homotopy equivalence. An equivalence between two objects that preserves their essential topological features.

Homotopic maps. Two maps,  and  , are homotopic if there exists a continuous map  (here  is a set of pairs  where  and  ) such that  and  .

Manifold. A topological space which at each point is topologically equivalent with Euclidean space  .

Multiplicativity of the Euler characteristic. The Euler characteristic is multiplicative in the sense that for two topological spaces *X* and *Y* for which the Euler characteristic is defined and such that there exists a covering  of finite degree *d* it follows that  .

Orbifold. A space which looks in most places like Euclidean space and has special points with a non-trivial symmetry group.

Polytope. A figure analogous to a polyhedron but defined in a space of an arbitrary dimension  . For  it is a point, for  a line segment and for  a polygon.

*k*-Simplex. A *k*-dimensional analog of a triangle.

Tessellation. A space-filling pattern.

Valuation. A function which satisfies the inclusion–exclusion principle.
